# Comprehensive Review on Zeolite-Based Nanocomposites for Treatment of Effluents from Wastewater

**DOI:** 10.3390/nano12183199

**Published:** 2022-09-14

**Authors:** Veena Sodha, Syed Shahabuddin, Rama Gaur, Irfan Ahmad, Rajib Bandyopadhyay, Nanthini Sridewi

**Affiliations:** 1 Department of Chemistry, School of Technology, Pandit Deendayal Energy University, Knowledge Corridor, Raisan, Gandhinagar 382426, Gujarat, India; 2 Department of Clinical Laboratory Sciences, College of Applied Medical Sciences, King Khalid University, Abha 61421, Saudi Arabia; 3 Department of Maritime Science and Technology, Faculty of Defence Science and Technology, National Defence University of Malaysia, Kuala Lumpur 57000, Malaysia

**Keywords:** zeolite, wastewater treatment, photocatalysis, nanocomposites

## Abstract

All humans and animals need access to clean water in their daily lives. Unfortunately, we are facing water scarcity in several places around the world, and, intentionally or unintentionally, we are contaminating the water in a number of ways. The rise in population, globalization, and industrialization has simultaneously given rise to the generation of wastewater. The pollutants in wastewater, such as organic contaminants, heavy metals, agrochemicals, radioactive pollutants, etc., can cause various ailments as well as environmental damage. In addition to the existing pollutants, a number of new pollutants are now being produced by developing industries. To address this issue, we require some emerging tools and materials to remove effluents from wastewater. Zeolites are the porous aluminosilicates that have been used for the effective pollutant removal for a long time owing to their extraordinary adsorption and ion-exchange properties, which make them available for the removal of a variety of contaminants. However, zeolite alone shows much less photocatalytic efficiency, therefore, different photoactive materials are being doped with zeolites to enhance their photocatalytic efficiency. The fabrication of zeolite-based composites is emerging due to their powerful results as adsorbents, ion-exchangers, and additional benefits as good photocatalysts. This review highlights the types, synthesis and removal mechanisms of zeolite-based materials for wastewater treatment with the basic knowledge about zeolites and wastewater along with the research gaps, which gives a quality background of worldwide research on this topic for future developments.

## 1. Introduction

**Figure 1:**
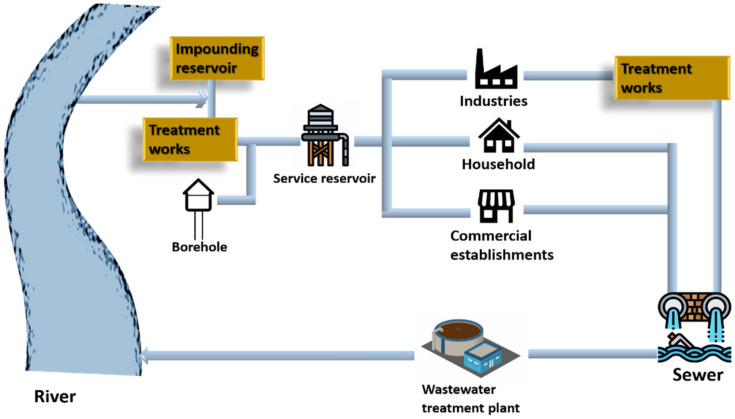
Cycle of water service.

Water is an integral part of all living organisms; it is crucial for humans and the environment. Water becomes contaminated after being used for numerous reasons such as bathing, washing, cooking, and manufacturing and is then dumped back into water sources after treatment. Since it is difficult for the wastewater treatment plants to treat the pollutants of emerging industries and the majority of industries levy fees for the same, it is less expensive for enterprises to treat or pre-treat the wastewater before discharging it into sewers. Figure 1 depicts how water is collected from resources, utilized, and then discharged into water bodies [1]. 

Wastewater can promote diseases such as polio, cholera, vomiting, diarrhoea, nausea, and even cancer in the human body [2,3]. If wastewater is discharged into water bodies, the pollutants in it can inhibit the establishment of marine plants [4]. There are numerous types of water pollutants for which multiple treatment methods have been described, which includes chemical, biological, and physical methods, such as adsorption [5], photocatalysis [6,7], ultrafiltration [8], and biofiltration [9]. In the area of wastewater treatment, zeolites, which are aluminosilicates with porous structures, are generally utilized as adsorbents [10,11], ion-exchangers [12], and photocatalysts [13,14]. The negatively charged structure of a zeolite attracts a variety of cationic pollutants to it. The use of zeolites in the removal of pollutants is not just restricted to the adsorption of cationic pollutants; by modifying it in various ways, its affinity for anionic pollutants can also be improved [15]. Photoactivity in zeolites has been enhanced by the addition of heteroatom to their framework, as in titanium silicates, exposing it to larger applications [16,17,18]. Composites are materials made by combining two or more materials known as parent materials. The term zeolite-based composite refers to the coupling of zeolites with other materials to form binary, ternary, and so on composites. The field of study on zeolite-based composites as a pollutant removal medium is broadening owing to their tuneable pore size [16,19], enhanced photoactivity [19], and easy operation [20]. For example, combining zeolites with materials that have a positively charged framework and an affinity towards anionic pollutants, may result in a composite that can be applied for the removal of both cationic and anionic contaminants. Generally, carbon-based materials [21,22], metal oxides [23,24], polymers [25,26], and clay compounds [27] are incorporated with zeolites for a variety of applications including fuel cells [28], catalysis [29], sorption [4], and others. Researchers have demonstrated the removal of contaminants from various model solutions, such as dyes [30,31], heavy metals [32], herbicides [33], etc., using zeolites and zeolite-based materials [34]. These models can also be applied to treat the real wastewater from industries. The synthesis, as well as adsorption and photocatalytic studies, on zeolite-based composites described in this review may help researchers in the treatment of real wastewater samples from industries. In addition, this review not only gives information about the zeolite-based composites, but also gives basic knowledge about wastewater and zeolites. The physicochemical properties of zeolites, synthesis of zeolite-based materials, and their mechanism in adsorption and photocatalysis are explained, which gives basic research background to early researchers and to scientists who aim to devise zeolite-based materials for pollutant remediation. The adsorption and photocatalytic research of zeolite-based composites are more thoroughly examined for the purpose of building a photoactive device for wastewater treatment.

### 1.1. Wastewater 

**Figure 2:**
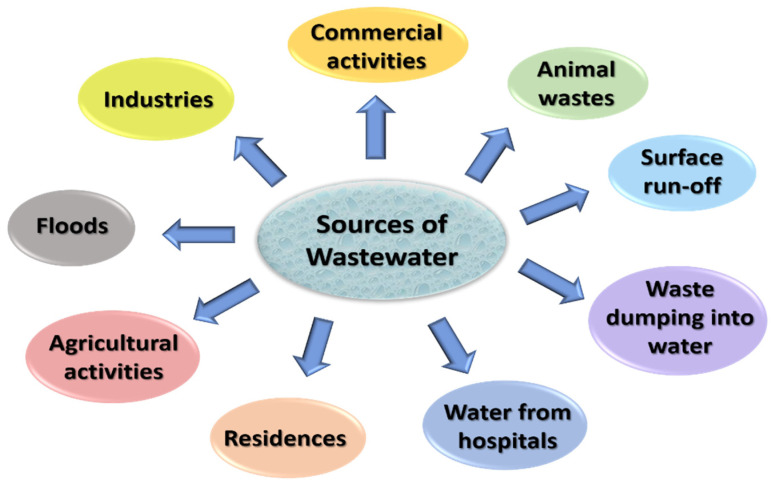
Sources of wastewater.

The combination of the waterborne or liquid wastes removed from institutions, residences, and industrial and commercial establishments is wastewater [35]. Everything that is discharged into the sewers subsequently gets treated in a wastewater treatment plant [1]. It includes pollutants from various domestic activities, such as bathing, cleaning clothes and utensils, and flushing toilets; industrial activities, such as textiles, mining, and manufacturing; commercial activities, such as beauty salons and car washing; agricultural facilities; energy units. Apart from these, a variety of other events, such as surface run-offs, floods, and storms also produce wastewater. Additionally, if the sewer becomes damaged, groundwater will sweep in, increasing the volume of wastewater. Figure 2 represents major sources of wastewater.

**Figure 3:**
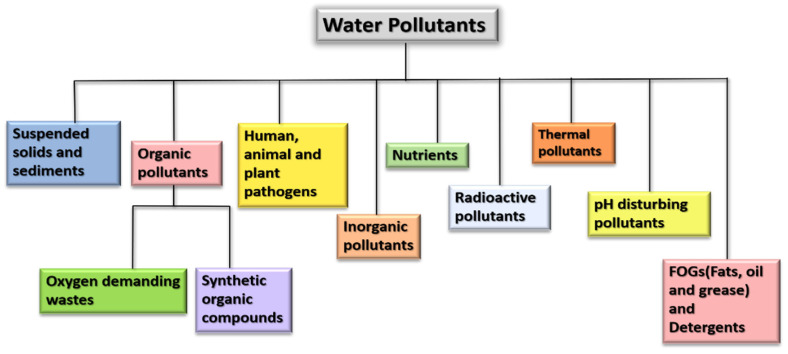
Flowchart representing various pollutants in wastewater.

Wastewater contains a variety of pollutants including organic, inorganic, toxic, non-toxic, thermal, and suspended solids from industries, residences, commercial activities, etc. [36]. Refer to Figure 3.

#### 1.1.1. Total Suspended Solids (TSS)

Suspended solids are particulate matter with a diameter of less than 62 µm. These are too small to settle down and too large to float, hence, they remain suspended in water. Generally, a water stream contains some SS, but an excessive amount might cause issues [37]. These exist in two forms: (i) inert and (ii) oxidizable solids. Sand particles and eroded minerals are examples of inert solids. These are sourced from mining, coal washing, construction sites, etc. Oxidizable solids settle out similar to inert solids but get decomposed on deposition releasing toxic compounds, such as methane, ammonia, and sulphides, causing higher oxygen demand in localized areas of water. These reduce the penetration of light into the water, inhibit the growth of filter feeders, cause temperature change, and other issues [1,37]. Their removal techniques include gravity settling [38], centrifugation [39], and filtration [40], followed by disinfection to remove floating bacteria and pathogens [41].

#### 1.1.2. Organic Pollutants

Organic pollutants can be classified based on two aspects: (i) the nature of the pollutant, i.e., natural or synthetic, and (ii) its persistence, i.e., a persistent or non-persistent organic pollutant. Natural organic pollutants include oxygen demanding wastes, which decrease the oxygen levels of water. Synthetic organic compounds include chemicals from industries, agricultures, etc. 

**Figure 4:**
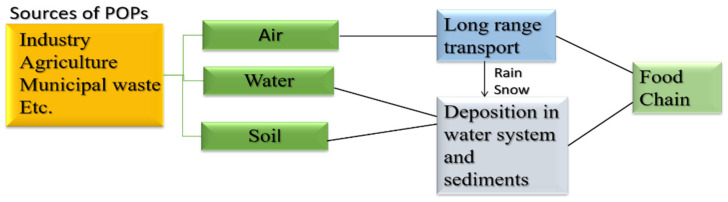
Flowchart representation of flow to POPs in the environment and food chain. Adapted with permission from Ref. [43]. Copyright 2020 Springer Nature.

Some organic pollutants are persistent and some are non-persistent. Persistent organic pollutants (POPs) are chemicals that live in the environment for prolonged periods as these are resistant to biochemical and photolytic processes [42]. Moreover, these are lipophilic and hydrophobic pollutants that are receptive to long-range transport and bioaccumulation and are prone to enter the food chain as well [43]. Consider Figure 4 [43].

Locally discharged POPs spread pollution far from its source. Sources of persistent organic pollutants (POPs) include volcanic activity and forest fires that produce dioxins and dibenzofurans. Other sources include agricultural pesticides and industries, such as dichlorodiphenyltrichloroethane (DDT), polychlorinated biphenyls (PCB), perfluoro octane sulfonic acid (PFOS), perfluorooctanoic acid (PFOA), and brominated flame retardants, etc. [44]. In adult studies, POPs have been implicated in a variety of adverse health impacts, such as thyroid and endocrine-related cancers, diabetes¸ obesity, and reproductive concerns in both males and females [45].

#### 1.1.3. Inorganic Pollutants 

Heavy metals, inorganic salts, mineral acids, trace elements, metals, and their complexes with organic compounds are examples of inorganic pollutants. Metals with a density higher than 5 g/cm^3^ are classified as heavy metals [46]. Titanium, cobalt, manganese, iron, nickel, nickel, copper, zinc, arsenic, silver, gold, and mercury are commonly occurring heavy metals in everyday life. Few heavy metals are essential elements in our life but when present in a large amount they can be toxic. Natural deposits of heavy metals can be discovered in the Earth’s crust, hence, one of their sources in wastewater includes surface run-off. Apart from that, metal-based industries, automobiles, roadworks, and metal leaching are the major sources of heavy metals in wastewater. Heavy metal exposure in humans can result in cellular function loss, cell damage, and potentially carcinogenic effects [46]. 

#### 1.1.4. Radioactive Pollutants

Approximately 11% of the world’s electricity is generated by nuclear power plants [47]. The nuclear fission process produces no carbon dioxide, which is a plus, but we still have to deal with nuclear waste. The release of radionuclides in the environment caused disaster during 1986 in the Chernobyl and Fukushima Daiichi plant in 2011 [48]. Radioactive pollutants can cause plant mutations and serious health damage to aquatic life. Their influence on humans may be mild or fatal depending on the magnitude and duration of exposure. When humans have a short time of exposure to a lower level of radioactive pollutants, it can cause mild skin irritations; on the other hand, prolonged exposure at low-intensity causes diarrhea, nausea, vomiting, hair loss, etc. Prolonged exposure to high levels of radiation will lead to some irreversible DNA damages. Apart from that, it can cause several other diseases, such as lung, thyroid, and skin cancers [49]. Several removal methods, including physical, chemical, and biological, are used. (i) Physical methods: evaporation, distillation, dumping; (ii) chemical methods: acid digestion wet oxidation, precipitation; (iii) biological: microbial remediation and plant remediation [3]. For further understanding of various types of pollutants refer to Table 1.

### 1.2. Zeolites

**Figure 5:**
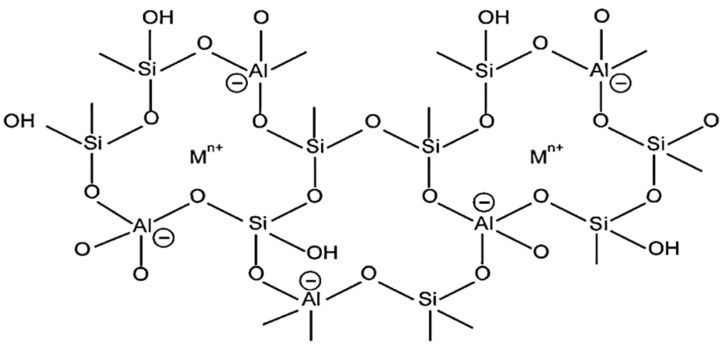
A two-dimensional illustration of the zeolite framework. Reprinted with permission from Ref. [62]. Copyright 2006 Elsevier.

Zeolites are crystalline three-dimensional, porous, aluminosilicates with organized structures and building blocks of tetrahedral units TO_4_ with an O atom bridged between them, as given in Figure 5, where T denotes Si or Al atom [61]. In the zeolite framework, the gap between the huge cavities holds water and interchangeable cations [15]. The basic chemical formula for zeolites is represented as:

$$M_{a/n}\left[{\mathrm{Al}}_{a}{\mathrm{Si}}_{b}O_{2\left(a+b\right)}\right]\cdot {\mathrm{qH}}_{2}O;$$

where M stands for [Sr, Ba, Ca, Mg] and/or [Li, K, Na], and cation charge is symbolized by n. The values of b/a range from 1 to 6 while q/a range from 1 to 4 [15]. 

Zeolites occur naturally as well as being synthesized chemically. The source of natural zeolites is volcanogenic sedimentary rocks. Clinoptilolite, phillipsite, mordenite, chabazite, stilbite, analcime, and laumontite are abundant among natural zeolites, while barrerite, offerite, and paulingite are rare. 

Some of the natural zeolites with their chemical formula are given in Table 2.

#### Classification of Zeolites

**Figure 6:**
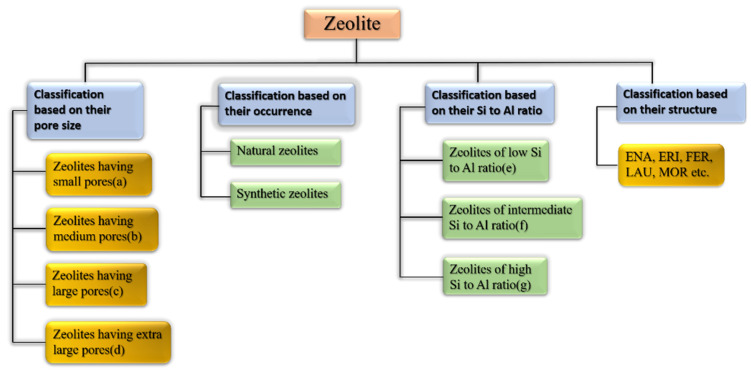
Broad classification of zeolites.

Zeolites may be categorized as per their occurrence, Si-Al ratio, pore size, crystal structure, and other factors [63]. Figure 6 shows the broad classification of zeolites. Table 3, Table 4 and Table 5 show how zeolites are classified according to their pore size, silica to alumina ratio, and structure type [63,64]. Some of the many classes of framework types according to the website of international zeolitic association (IZA) are depicted in Table 5.

Catalysis, cation exchange, sorption, and molecular sieving are all physiochemical characteristics of zeolites. The tetrahedrons of [SiO_4_]^4−^ and [AlO_4_]^5−^ are linked together in the zeolitic framework to build cages linked via precise and molecular-sized pores. Existence of [AlO_4_]^5−^ gives a negative charge to zeolite structure, which is stabilized by positively charged ions, such as K^+^, Na^+^, and Ca^2+^. These ions are responsible for ion-exchange processes in zeolites [15]. The porous structure of a zeolite has been proven to have excellent adsorption efficiency for heavy metals, including mercury, fluoride, arsenic, and organic dyes.

### 1.3. Adsorption

One of the most common wastewater treatment methods is adsorption since it is simple to use and effective [65]. It is a surface phenomenon that occurs when the molecules from fluid bulk come into contact with a solid surface either by physical forces or chemical bonds. Usually, adsorption is a reversible process. Reversible of adsorption is when the adsorbent begins to release the adsorbed molecules; this is referred to as desorption [66]. There are two types of adsorptions: (i) physical adsorption, i.e., adsorption under the influence of physical forces such as weak van der Waals attractions, hydrogen bonding, etc., and (ii) chemical adsorption, i.e., adsorption by chemical bonds [67].

**Figure 7:**
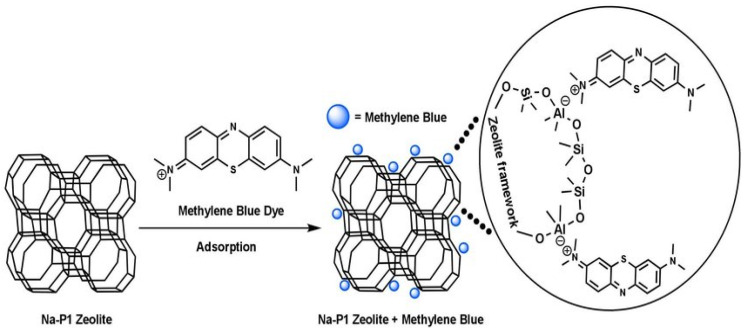
Adsorption of methylene blue on Na-P1 zeolite. Reprinted from Ref. [10].

Activated carbon [68], industrial solid wastes [69], biomaterials [70], clay minerals [71], and zeolites [11,14] are among the most commonly used materials in wastewater treatment. In the year 1785, adsorption was first discovered by Lowitz, after that, it was used in sugar refining processes to remove color [72]. Subsequently, in American treatment plants, inactivated charcoal filters were employed for water purification [72]. For the first time, granular activated carbon (GAC) was used for adsorption in 1929 in Hamm, Germany, and Bay City, Michigan, 1930 [73]. When studied, modified and synthetic zeolites showed better adsorption and ion exchange capacities among synthetic, modified, and natural zeolites [72]. Zeolites are mostly used for the adsorption of heavy metals [32], dyes [74], ammonium ions [75], etc. Natural turkey clinoptilolite exhibited low absorptivity for three azo dyes (Everzol black, Everzol red, Everzol yellow) as examined by Armagan et al. [76]. Adsorption capacities of natural zeolite were improved greatly via modification with quaternary amines [76]. Figure 7 elaborates on the adsorption of methylene blue on zeolites which occurs via electrostatic attractions between negatively charged Al ions in the zeolite framework and positively charged nitrogen atom in methylene blue.

### 1.4. Photocatalysis

**Figure 8:**
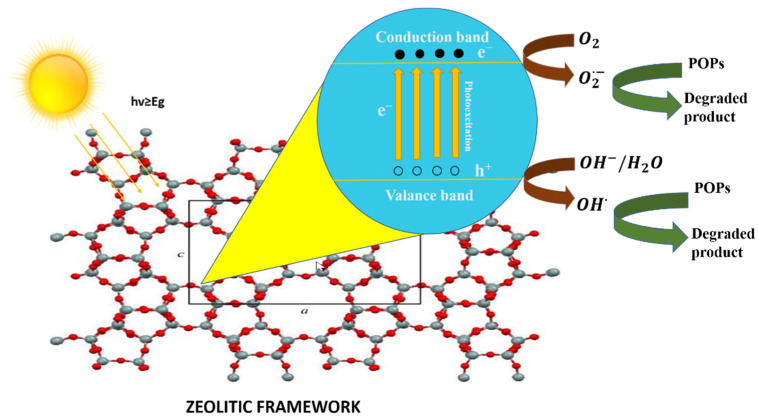
Schematic diagram of photocatalysis in zeolite (image of zeolite framework is reprinted from Ref. [78]).

Photocatalysis is the process in which a photon of light catalyzes a reaction. Materials with photocatalytic characteristics are known as semiconductors [77]. The conduction band and valance band are two different energy bands in a semiconductor. The bandgap is the energy difference between the two above-stated bands. When a photon of light strikes a semiconducting material, electrons (e^−^) are excited from the valance band to the conduction band and positively charged holes (h^+^) are left behind, as shown in Figure 8. The photo-generated electron-hole pairs develop the active oxidizing species, which then cause organic pollutants, such as dyes, in wastewater to degrade [43]. 

Photocatalysis can be categorized into two types: (i) homogenous and (ii) heterogeneous photocatalysis. If in a photocatalytic reaction, both photocatalyst and reactant are in a different phase, then it is said to be heterogeneous photocatalysis. On the other hand, if photocatalyst and reactants and all other species, such as photosensitizers, are in the same phase, the system is called homogenous photocatalysis [79]. Due to its great stability, ease of separation, and photocatalyst regeneration, heterogeneous catalysis is superior to homogeneous catalysis [80].

The following steps are part of the heterogeneous photocatalysis mechanism [43].

$$\mathrm{Semiconductor}\;\mathrm{photocatalyst}\;\overset{\mathrm{hv}\geq E_{g}}{\to }{\;e}^{-}+h^{+}\;\;h^{+}+H_{2}O\to {\mathrm{OH}}^{•}+H^{+}\;h^{+}+{\mathrm{OH}}^{-}\to {\mathrm{OH}}^{•}\;e^{-}+O_{2}\to O_{2}^{•-}\;O_{2}^{•-}+H^{+}\to {\mathrm{OOH}}^{•}\;2{\mathrm{OOH}}^{•}\to H_{2}O_{2}+O_{2\;}\;H_{2}O_{2}+O_{2}^{•-}\to {\mathrm{OH}}^{-}+{\mathrm{OH}}^{•}+O_{2}\;\mathrm{POPs}+\left(h^{+},{\mathrm{OH}}^{•},O_{2}^{•-},{\mathrm{OOH}}^{•}\mathrm{or}\;H_{2}O_{2}\right)\to \mathrm{Degraded}\;\mathrm{products}\;$$

Photocatalytic activities of zeolite alone are rarely discussed. Research on the photocatalytic activity of zeolites is scarce. Rather, their cavities behave as hosts for semiconducting materials in photocatalysis. In 2003, Krisnandi et al. investigated zeolite ETS-10, a titanosilicate, microporous zeolite with liner Ti-O-Ti-O-chains in the framework as a photocatalyst for oxidation of ethane to CO_2_ and water [79]. Before this work, some preliminary analyses have been conducted on the photocatalytic activity of ETS-10 [81]. The basic mechanism in ETS-10 is that the titanium sites present in Ti-O-Ti-O trap the electrons and undergo photoreduction in the existence of ethane. These trapped electrons quickly get shifted to oxygen and generate active oxidation species [79]. In 2020, Aguiñaga et al. reported clinoptilolite–mordenite, natural zeolite as an efficient self-photocatalyst [13]. The obtained results were compared with that of titanium dioxide particles, and it was found that under similar conditions, both zeolite and TiO_2_ required the same time for the complete degradation of caffeine.

**Figure 9:**
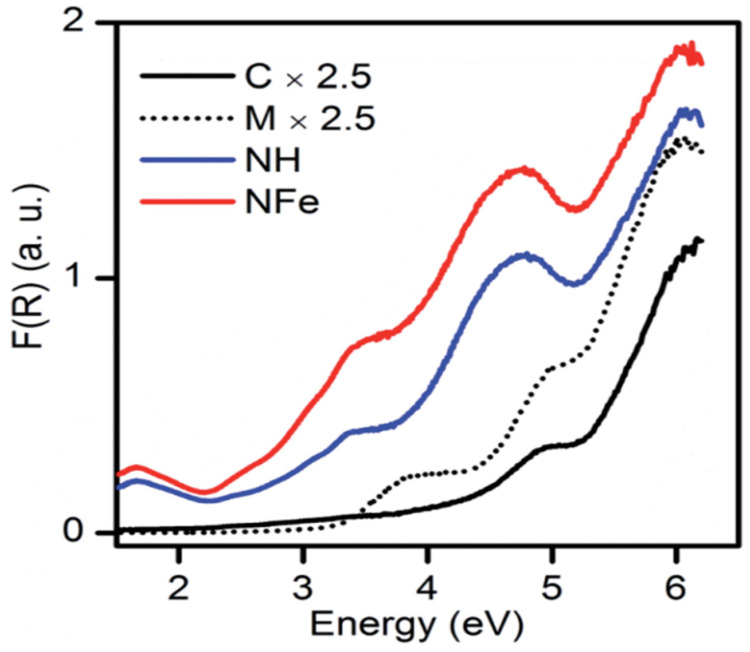
Diffuse reflectance spectra of synthetic and natural zeolites. Reprinted from Ref. [13].

The NH abbreviation is used for the hydrogenated form of natural zeolite (NZ) and NFe denotes the ion-exchanged version of NZ. The symbol SH stands for synthetic zeolite clinoptilolite–mordenite. Aguiñaga et al. recorded the diffuse reflectance spectra of synthetic and natural zeolites as given in Figure 9. A bandgap analysis was conducted with the Tauc plot. Based on spectroscopic analysis of ZSM-5, it was estimated that bands developed in the range from 200–500 nm are attributed to different states of iron, and in synthetic zeolites, the presence of iron is usual. The aluminosilicate framework has a bandgap of approximately 7 eV. The bandgap of natural mordenite was estimated as 2.63 eV. In the case of synthetic clinoptilolite C, the analyzed band gap was 4.26 and 4.46 eV for direct and indirect transitions, respectively. In the same way, for synthetic mordenite, it was estimated at 3.26 and 3.45 eV. Hence, the wide bandgap of zeolite resembles the semiconductors and its application as a photocatalyst can be of interest in future research [13].

### 1.5. Ion Exchange

**Figure 10:**
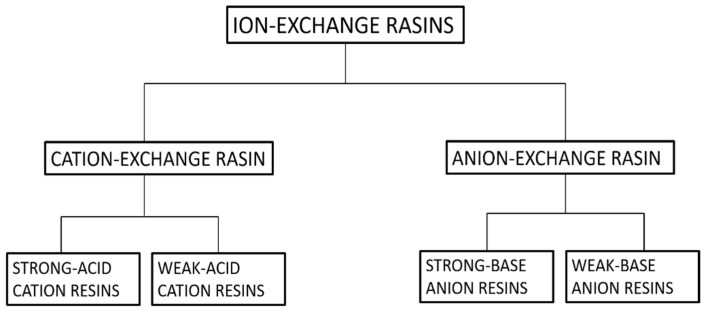
Classification of ion-exchange resins.

Ion exchange can be defined as a reversible process in which exchangeable ions in an insoluble exchange material replaces similarly charged ions in the solution [82]. Electrostatic attractions are employed between ionic functional groups, which is the driving force of a typical ion exchange reaction. It can easily be employed for the extraction of heavy metals, such as cadmium, chromium, barium, arsenic, silver, lead, as well as nitrates from water [83]. Furthermore, ion exchange is the best process for the removal of radioactive nuclide in small systems [84]. Ion-exchangers can be categorized as cation and anion exchange resins. Cation exchangers are those that interchange their cations, and anion exchangers interchange their anions with the solution [84]. For further clarification refer Figure 10.

**Figure 11:**
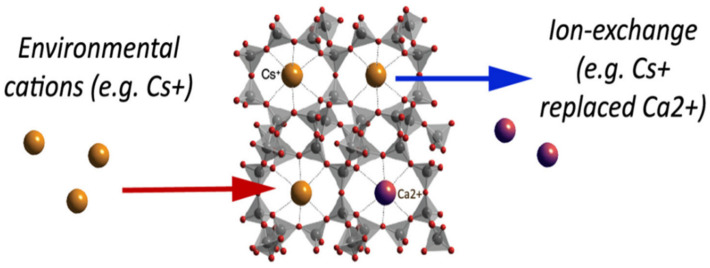
Ion exchange in naturally occurring zeolite, clinoptilolite. Reprinted from Ref. [89].

Natural inorganic zeolites, clays, and synthetic organic resins are extensively used ion-exchangers [85,86]. One of the major drawbacks that are associated with ion-exchangers is that their ion-exchange capacity is easily reduced with contamination via organic substances. Luca et al. used ETS-10 zeolite to remove metals originating from zinc ferrite [12]. A titanosilicate, ETS-10, is microporous zeolite with liner Ti-O-Ti-O-chains in its framework. ETS-10 is extensively employed as an ion-exchanger [87]. The major advantage of using ETS-10 as an ion-exchanger is that it is easy to regenerate and is thermally stable up to 550 °C [88]. Hence, if contaminated with organic pollutants, it can easily be regenerated by calcination. Based on ICP-MS elemental analysis, it was estimated that zinc ferrite releases high concentrations of Fe, Zn, Pb, Ca, and Mn. ETS-10 removed all metal ions present very efficiently. ETS-10 showed better cation exchange capacity (CEC) than commercial zeolite A for manganese, zinc, and lead. Nearly 100% removal was observed within 30 min [12]. Figure 11 gives a basic idea about how the ion-exchange mechanism works in zeolites. A natural zeolite, clinoptilolite, which has Ca^+2^ ions trapped in its cavity, is used as an ion-exchanger. Ca^+2^ ions are replaced with Cs^+2^ via ion exchange as depicted in Figure 11.

The adsorption properties of zeolites are influenced by their chemical and structural makeup. The cation exchange capacity (CEC) of zeolites varies with zeolitic framework structure, the density of anionic framework, size, and shape of foreign ions, etc. In raw natural zeolites, the pores are clogged with impurities and there is no uniform pore distribution, crystal structure, or chemical composition throughout the framework [90]. Additionally, raw natural zeolites have a negatively charged surface, hence, they only attract cationic pollutants, such as cationic dyes and heavy metal ions. They have a very low or little affinity toward anionic pollutants, such as anionic dyes and organic pollutants in aqueous media. Their efficiency can be increased by their modification. Table 6 shows some of the modification processes [34,91].

We observed that raw, natural zeolites have limited adsorption capacity and carry contaminants. Synthetic zeolites, on the other hand, have a consistent pore distribution across the framework, as well as improved adsorption and ion exchange behavior. Kozera-Sucharda et.al. conducted an experiment on the removal Cd^+2^ and Pb^+2^ by natural and synthetic zeolites [98]. They witnessed faster and efficient removal of Cd^+2^ and Pb^+2^ from multicomponent solutions with synthetic zeolites [98]. Zeolites can be synthesized by using raw natural materials, such as natural silica sources, as well as using synthetic precursors as well. Zeolite produced using natural precursors is inexpensive but lacks precise pore structures and contains contaminants. Zeolite produced from synthetic precursors has a precise structure and fewer imperfections but is expensive.

The challenge of reusability with nanosized synthesized zeolites is another reason that reduces their effectiveness. Zeolites demonstrate improved physiochemical stability, greater adsorption capacity, and simpler reusability when utilized in the form of their composites. Additionally, the characteristics of the materials used to create composites have significant advantages of their own. Most frequently, it has been found that the zeolite-based composites show better optical properties and pore distributions than zeolites themselves, increasing the application of zeolites in adsorption, photocatalysis, and other processes. Zeolites function in photocatalytic reactions in two ways: either as a host for semiconducting materials or by cooperating with those materials’ electron transfer processes to significantly reduce the chance of electron–hole recombination.

## 2. Zeolite-Based Composites

In general, zeolite-based composites for pollutant removal from wastewaters are made by incorporating metal oxide nanoparticles, carbon-based materials, clay compounds, and polymers into zeolites. 

### 2.1. Synthetic Approaches

**Figure 12:**
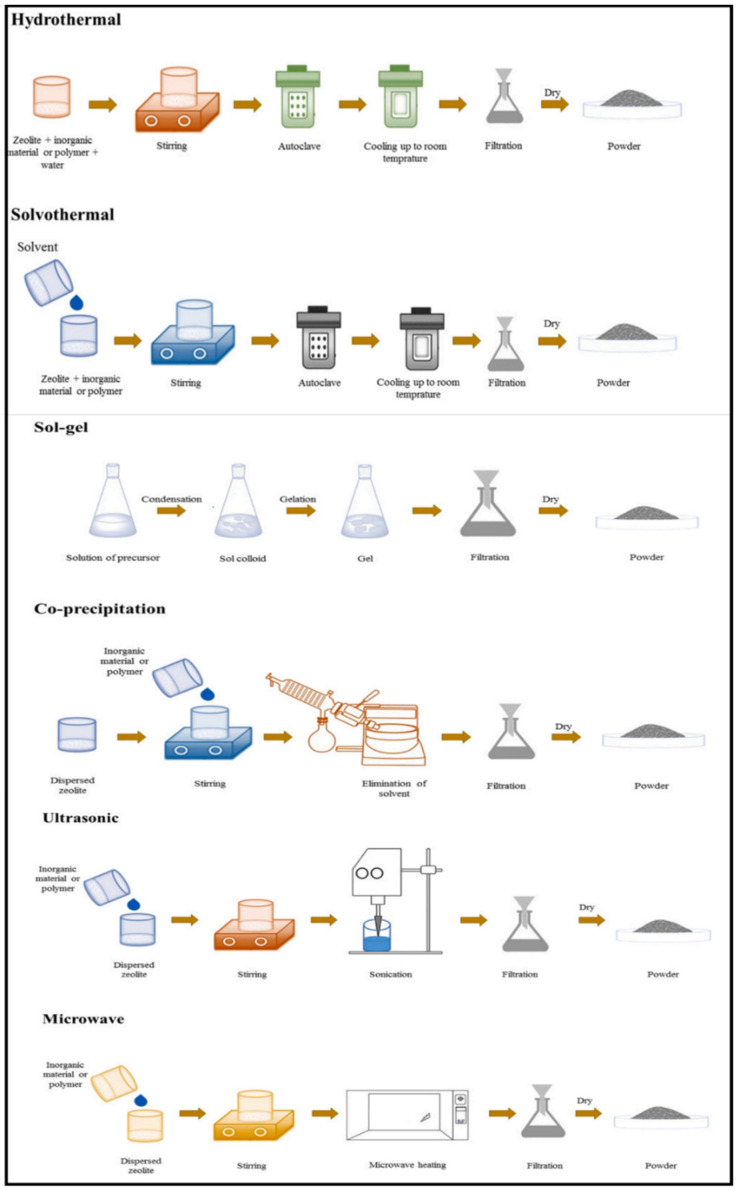
Synthesis processes of zeolite/metal oxide composites. Reprinted with permission from Ref. [34]. Copyright 2021 Elsevier.

General methods for the synthesis of zeolite/metal oxide composites are (i) sol-gel, (ii) hydrothermal, (iii) solvothermal, (iv) co-precipitation, (v) ultrasonic, and (vi) microwave. Figure 12 summarizes the techniques used for the preparation of zeolite/metal oxide composites [34].

**Figure 13:**
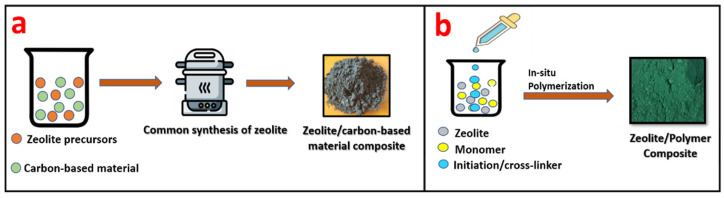
Schematic representation of (**a**) the synthesis of a zeolite/carbon-based material composite and (**b**) a zeolite/polymer composite.

Zeolite/carbon-based materials are generally synthesized by the conventional synthesis method of zeolites, such as hydrothermal, solvothermal, sol-gel, etc. During the initial stage in the synthesis of zeolites, carbon-based material is added along with the precursors of zeolites, leading to the formation of a zeolite/carbon-based material composite, as given in Figure 13a.

Zeolite/polymer composites are generally fabricated by in situ polymerization. In this process, monomer and zeolite are mixed together followed by polymerization, which leads to the formation of a zeolite/polymer composite. Figure 13b shows the schematic of the synthesis of a zeolite/polymer composite. 

### 2.2. Removal Process

**Figure 14:**
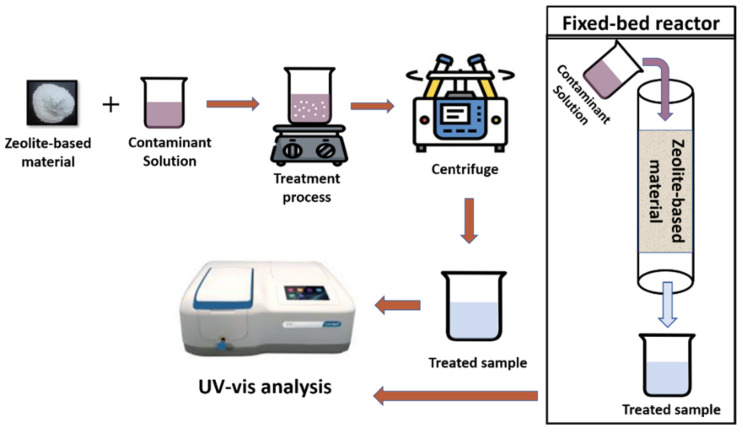
Schematic representation of pollutant removal process using zeolite-based materials.

When working with zeolites and zeolite-based composites, adsorption, ion-exchange, and photocatalysis are the general pollutant removal processes in wastewater treatment. In these processes, a known amount of catalyst/adsorbent is added into the model contaminant solution, i.e., dyes, heavy metals, agrochemicals, etc. Then, this suspension is kept under the treatment process and treated samples are centrifuged and analyzed using a UV–Vis spectroscope. Zeolite-based materials are also used in the fixed-bed reactors to remove pollutants from water as depicted in Figure 14.

### 2.3. Zeolite/Metal Oxide Composites

Metal oxides are employed in a variety of applications, such as adsorption, photocatalysis, energy storage, etc., due to their tunable size and morphology [99]. When employed in their nano form, metal oxides have a large surface area and exhibit excellent adsorption capabilities. Metal oxides are an ideal photocatalyst due to their distinct physicochemical properties, which include shape, size, morphology, composition dependence, and light sensitivity [100]. When doping metal oxides with zeolites, the cavities of zeolites behave as a support for metal oxides, increasing the surface area of metal oxides, thereby improving adsorption and photocatalytic properties.

**Figure 15:**
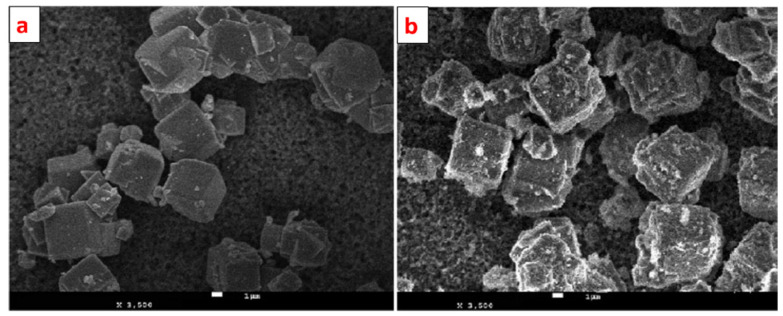
FE-SEM images of (**a**) zeolite and (**b**) ZnO nanoparticle-doped zeolite. Reprinted from Ref. [101].

Alswata et al. synthesized zeolite/ZnO nanocomposites using the co-precipitation method. Prepared samples were examined for the adsorption of lead Pb(II) and arsenic As(V) from its synthetic solution [101]. The FE-SEM images of bare and ZnO-doped zeolite are given in Figure 15. From the FE-SEM, it is evident that zeolite has a cubic shape with a smooth surface, while some granular doping can be observed on the surface of ZnO-doped zeolite. Zeolite’s cubic shape remains as it is after doping of ZnO NPs.

Under similar experimental conditions, the zeolite/ZnO nanocomposite showed better adsorption capacity than zeolite alone for the removal of both arsenic (As) and lead (Pb). When combined with ZnO, zeolite eliminated 92% of the lead and 85.7% of the arsenic, respectively, compared to 43.6% and 32.3% for pure zeolite [101].

Sacco et al. integrated semiconducting ZnO into zeolite cavities by wet impregnation method and prepared ZnO/ZeO pellets [24]. These pellets were evaluated for the removal of caffeine by a simultaneous process of photocatalysis and adsorption. The studies were performed in two stages, i.e., by adsorption and by adsorption assisted photocatalysis. At the initial stage, the adsorption kinetics were found to be faster in the case of zeolite as compared to the composite. This might be due to decreased mesoporous surface area of the composite, though, the total adsorption was approximately 60% for both the ZnO and ZnO/ZeO composite. When the removal of caffeine by adsorption and adsorption/photocatalysis were examined, it was shown that UV irradiation resulted in a significantly higher total removal of caffeine. Almost 100% of caffeine was removed within 120 min of reaction time by using the ZnO/ZeO composite in adsorption/photocatalysis, while only adsorption gave 69% removal in 120 min. Under UV irradiation, ZnO can degrade the adsorbed caffeine and its chemical intermediates, creating active sites for the adsorption of any leftover caffeine molecules in the liquid medium [24].

Mahalakshmi et al. fabricated the zeolite-supported TiO_2_ composite by using the H-form of zeolite Y, β, and ZSM-5 and labeled them as HY, Hβ, and H-ZSM-5, respectively [102]. The prepared materials were investigated for adsorption and photocatalytic degradation of propoxur, an *N*-methylcarbamate pesticide. According to the experimental data, the adsorption of propoxur was better over TiO_2_/Hβ than HY/ TiO_2_ and H-ZSM-5/ TiO_2_. Propoxur degradation efficiency was found to be better in TiO_2_/Hβ with optimal TiO_2_ loading (7 wt%) than in pure TiO_2_. The limited surface area of H-ZSM-5 and the hydrophilic character of HY were responsible for their poor adsorption capacity. The existence of acid sites in Hβ with high acid strength might be another factor in propoxur adsorption [102].

**Figure 16:**
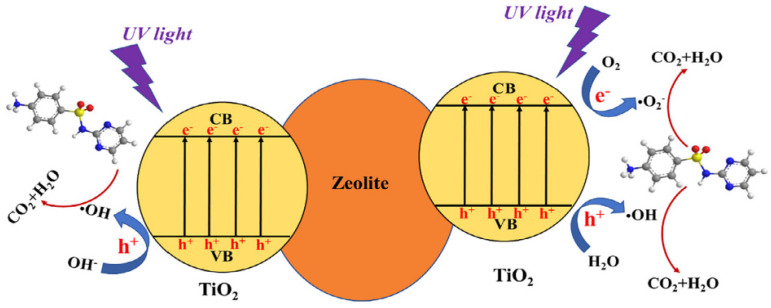
Mechanism of sulphadiazine removal by using zeolite/TiO2 composite. Reprinted with permission from Ref. [23]. Copyright 2018 Elsevier.

Liu et al. evaluated the TiO_2_/zeolite composite for the removal of sulfadiazine (SDZ) via adsorption and photocatalysis under UV light [23]. The composite material was synthesized via the sol-gel method. FTIR analysis indicated the formation of the Ti-O-Si bond in the composite. In 60-min dark studies performed, a small amount of adsorption of SDZ was reported. UV light studies showed the 32.76% degradation of SDZ in 120 min without the aid of a catalyst. The prepared TiO_2_/zeolite composite removed 93.31% of SDZ in the presence of UV light within 120 min. The general mechanism of degradation is as given in Figure 16 [23].

The participation of reactive oxygen species (ROS), i.e., $e^{-}$, $h^{+}$, ${\mathrm{OH}}^{•}$, $O_{2}^{-•}$, ^1^O_2_ (singlet oxygen), were examined by performing the scavenger studies. It was concluded that ROS contribution in the zeolite/TiO_2_ composite follows the order of ${\mathrm{OH}}^{•}\;$>${\;h}^{+}$ >${\;O}_{2}^{-•}\;$> ^1^O_2_. Additionally, the HPLC–MS/MS study of reaction intermediates was used to hypothesize the four potential degradation pathways [23].

D Mirzaei et al. synthesized the NaX/MgO–TiO_2_ zeolite nanocomposite by using the ultrasound-assisted dispersion method [103]. Anionic dye methyl orange was used to investigate adsorption on the prepared composites. To obtain maximum MO adsorption yield from the aqueous solution, different parameters such as initial dye concentration, adsorbent dosage, pH, adsorbent type, and contact time have been assisted and optimized. To estimate the adsorption–desorption isotherm, Freundlich, Temkin, and Langmuir models were used. For subsequent processes, chemical parameters such as 0.3 g L^−1^ adsorbent dosage, 6.5 pH, contact time of 35 min, and temperature of 45 °C were evaluated as the optimized conditions. Under identical experimental conditions, it is determined that the NaX/MgO–TiO_2_ nanocomposite led to the highest 95% adsorption efficiency from aqueous solution among NaX, MgO, TiO_2_, MgO–TiO_2_, and NaX/MgO–TiO_2_ adsorbents, and MO adsorption efficiencies over MgO, TiO_2_, NaX, MgO–TiO_2_, and NaX/MgO–TiO_2_ were greater than 30%, 46%, 40%, 68%, and 95%, respectively [103].

A.A. Alswat et al. used a co-precipitation approach to make zeolite/iron oxide (Fe_3_O_4_) and zeolite/copper oxide (CuO) nanocomposites (NCs) [104]. The adsorption efficiencies were 97.2% and 96.8% for Pb and As, respectively, by zeolite/iron oxide (Fe_3_O_4_) NCs, and 83.7% and 81.3% for Pb and As, respectively, by zeolite/ copper oxide (CuO) NCs at a pH of between 4 and 6 when these composites were kept for 40 min at room temperature and pressure. The Langmuir isotherm model was well followed by the adsorption data [105]. 

Kong et al. used the co-precipitation method to prepare nanosized Fe-Al bimetallic oxide-doped zeolite spheres and used it to remove Cr(VI) ions from constructed wetlands [106]. The pseudo-second order model was found to be perfectly fitted to the removal of Cr(VI). The composite zeolite spheres outperformed standard fillers in terms of removal, with excellent adsorption across a wide pH range. The Cr(VI) was absorbed and fixed by the composite zeolite spheres, and then it was reduced to Cr(III) using the Fe-Al oxide. Through co-precipitation and ion exchange, the Cr(III) made Cr(OH)_3_ and Cr_x_Fe_1-x_(OH)_3_ precipitates [106]. 

Zhang et al. used a one-step hydrothermal method to fabricate TiO_2_/MoS_2_ photocatalysts supported on zeolite utilizing micrometer-MoS_2_ as the sensitizer [107]. Under simulated solar-light irradiation, the synthesized photocatalyst TiO_2_/MoS_2_/zeolite had significantly higher photocatalytic response than the Degussa P25 photocatalyst. The recombination of photogenerated electrons and holes is one of the major factors that limit the efficiency of a photocatalyst. According to Zang et al., during the fabrication procedure, the Z-scheme photocatalyst of TiO_2_/MoS_2_ was developed where MoS_2_ acted as an electron donor in interfacial charge conduction, thereby improving the charge separation. In addition, the generation of superoxide anion radicals ($O_{2}^{-•}$), major oxidation species in photocatalytic reactions, can be aided by the micro/nano-MoS_2_ generated via the hydrothermal process [107]. 

D. Wang et al. successfully prepared Cr-doped TiO_2_ photocatalysts supported on natural zeolite [104]. Because Ti^4+^ and Cr^3+^ have similar ionic radii, Cr ions can be integrated into the TiO_2_ lattice by taking the place of Ti^4+^ sites. As the calcination temperature rises, the bond strength of Cr-O-Ti increases. Cr dopant is found as Cr^6+^ (81.2%) and Cr^3+^ (19.8%) species. In comparison to undoped TiO_2_/zeolite, the band gap energy (eV) of 10 mol% Cr/TiO_2_/zeolite decreases dramatically from 2.84 eV to 1.70 eV. The percentage degradation of methyl orange by the calcined 10% Cr/TiO_2_/zeolite reaches 41.73%, after 5 h of illumination, which is 17.9% higher than the degradation efficiency of undoped TiO_2_/zeolite [104].

Italia et al. prepared two composites of zeolite and bentonite separately bonded with titanium and was evaluated for the adsorption of phosphate. Titanium/zeolite and titanium/bentonite composites removed up to 83% and 84% of phosphate at 3 pH [108].

However, it is not always observed that the composites exhibit superior removal efficiencies than the zeolites alone. A. Alcantara-Cobos et al. compared ZnO nanoparticles and the ZnO-zeolite composite for tetrazine removal [20]. The zeolite-ZnO composite was prepared by the chemical precipitation method. Both adsorption and photocatalysis were working mechanisms behind the removal of tetrazine. The adsorption with ZnO nanoparticles was faster than the ZnO-zeolite composite. The degradation reported followed by adsorption under UV light radiations was 81% and 87% for the zeolite-ZnO composite and ZnO nanoparticles, respectively; although, the latter was difficult to remove from the aqueous solution. Additionally, the ZnO nanoparticles show low toxicity towards Lactuca sativa when kept with the dye solution and diluted aqueous solutions [20]. 

Jaramillo-Fierro et al. synthesized extruded semiconducting ZnTiO_3_/TiO_2_ supported on zeolite and its precursor clay [109]. Zeolites were synthesized by using two types of Ecuadorian clays via hydrothermal treatment and the method of alkali fusion, i.e., R-clay and G-clay. Zeolite prepared using R-clay was labeled as R-zeolite and was mostly of the Na-LTA type with a trace quantity of Na-FAU type. Zeolite prepared using G-clay was labeled as G-zeolite and was made up primarily of Na-FAU type zeolite with residues of Na-P1 type zeolite. The semiconducting support ZnTiO_3_/TiO_2_ was prepared separately via the sol-gel method. The composites were prepared by mixing zeolites, precursor clays, and ZnTiO_3_/TiO_2_ in different ratios. The reported order of adsorption of MB on parent material was G-zeolite > R-zeolite > G-clay > R-clay > ZnTiO_3_/TiO_2_. Despite having a higher dye removal capacity than mixed oxide ZnTiO_3_/TiO_2_ and precursor clays, the capacity of the extruded composites to remove MB was not increased by zeolites. Additionally, extruded zeolites are less capable of removing the color than powdered zeolites because they have a lower specific surface area [109]. 

### 2.4. Zeolite/Carbon-Based Material Composites

Currently, carbon nanomaterials are considered to be the most adaptable materials that can be employed to improve wastewater treatment techniques. Innovative carbon materials have been discovered as a result of extensive research conducted globally and effectively used in wastewater remediation and environmental safety technologies [110,111]. SWNT (single-walled carbon nanotubes), MWNT (multi-walled carbon nanotubes), G (graphene), and GO (graphene oxide) are among the most frequently studied carbon-based nanomaterials. These materials may be employed in their natural forms or as complex hybrid substances [111,112].

Zeolites have been modified by the addition of a heteroatom to their structural framework, giving them new and fascinating qualities, including photoactivity [17,18,113]. Ren et al. produced new generation photocatalysts by combining functional inorganic nanomaterials (such as zeolitic TS-1) with graphene and carbon-nanotube (CNTs) [16]. The performance of these photocatalysts were outstanding owing to (i) the synergistic effect on the basis of interfacial charge and heat transfer reactions and (ii) graphene’s ability to modify the shape and size of TS-1. The few layers of graphene were first synthesized via applying direct current discharge to graphene. Zeolite TS-1 was synthesized via the sol-gel process. Zeolite/graphene and zeolite/CNT hybrids were prepared by combining in situ to the common sol-gel synthesis of TS-1. The photocatalytic behavior of these materials was examined through dye degradation in the presence of low-intensity UV radiations. It was observed that the photocatalytic activity of TS-1 zeolite increased 27–28 times with graphene loading and only 4–5 times by CNT loading. The reason is the interfacial charge transfer between the conduction band of zeolite and nanocarbon. Moreover, the high reactivity of edge atoms in graphene might be responsible for high photocatalytic activity [16].

W.A. Khanday et al. synthesized a zeolite/activated carbon composite from oil palm ash using a two-step method, i.e., fusion followed by the hydrothermal process and labeled as Z–AC composite [114]. The adsorption results of MB adsorption on the Z–AC composite were compared with those of activated and non-activated oil palm ash and oil palm ash zeolite. The highest adsorption capacity observed for the Z–AC composite was approximately 90% [114].

H. Li et al. prepared zeolite by granulation, calcination of coal gangue, followed by the hydrothermal process and marked as ZMC [19]. Since the carbon content was removed at the calcination step, prepared ZMC is pure zeolite. By modifying the above-mentioned process, they synthesized a carbon retaining and extra carbon-containing zeolite-activated carbon composite and marked them as ZTC and ZAC, respectively. The prepared sample were examined for the adsorption of Cu^+2^ and rhodamine-B(Rh-B). The adsorption capacities for Cu^+2^ was observed to be 98.2%, 97.1%, 92.8% for ZMC, ZTC, and ZAC, respectively. Similarly, the adsorption capacities for RB were 17.0%, 41.3%, and 94.2% for ZMC, ZTC, and ZAC, respectively. Adsorption capacity for Cu^+2^ decreases upon increasing carbon content. Zeolite has a uniform pore distribution with a pore size of 0.41 nm, which can easily hold small Cu^+2^ ions. On the other hand, activated carbon has variable pore sizes across the network, including micro, meso, and macro pores; high pore sizes are incompatible with holding tiny Cu^+2^ ions and are suitable for adsorption of large organic molecules; hence, adsorption capacity for Rh-B increasing upon increasing carbon content [19]. 

M.A. Farghali et al. synthesized a mesoporous zeolite A/reduced graphene oxide nanocomposite [31]. For this, zeolite was first surface-modified with the help of 3-aminopropyl-trimethoxy silane (APTMS), which is used as a binding and mesopore generating agent. Then, using a hydrothermal technique, reduced graphene oxide was added to zeolite-A to create modified mesoporous zeolite-A/reduced graphene oxide NCs (MZ-A/RGO). Following that, the synthesized material was employed to remove lead ions (Pb^+2^) and methylene blue at the same time. The synthesized composite removed 98% of methylene blue and 93.9% Pb^+2^ [31].

Mahmoodi et al. immobilized the laccase enzyme onto a zeolite and graphene oxide composite via the covalent bond and prepared a biocatalyst for the removal of direct red 23 dye [22]. They used the hydrothermal method for the preparation of zeolite and the Hummers method for the preparation of graphene oxide. The composites were prepared by taking different weight ratios of graphene oxide followed by laccase immobilization. It was observed that the dye degradation increased upon increasing the loading ratio of graphene oxide. Graphene oxides increase the electron transfer between dye and enzyme, thereby enhancing the oxidation ability of the enzyme [22].

Huang T et al. evaluated magnetic graphene oxide-modified zeolite for uptake of methylene blue from an aqueous solution [21]. The magnetic MnFe_2_O_3_ nanoparticles were synthesized by the co-precipitation method. The Cu-zeolite was made separately as Cu-Z. The Cu-Z-GO-M composite was prepared using the solid-state dispersion method. The composite offered the highest adsorption capacity, i.e., 97.346 mg/g at 318 °C [21].

Using two-step alkali fusion and hydrothermal treatment, Zhao et al. generated a honeycomb-activated carbon-zeolite composite (CZC) using coal fly ash (FA) and utilized it to adsorb Pb^+2^ from an aqueous solution [4]. The pre-treated coal fly ash was heated in a muffle furnace to 750°C as part of the activation process to produce activated carbon. Activated carbon zeolite CZC owns a specific surface area that is approximately six times greater than FA’s, and its average pore size is enlarged from 3.4 to 12.7 μm. At pH 7, CZC demonstrated 185.68 mg/g of Pb(II) absorption after 40 min of contact time. According to kinetics studies, Pb(II) ion adsorption onto the surface of CZC is more consistent with pseudo-second order kinetics [4].

### 2.5. Zeolite/Polymer Composites

Polymers are the organic compounds with a variety of exceptional properties, including high mechanical strength, extraordinary flexibility, large surface area, and chemical stability. Owing to these characteristics, polymers can serve as a host for many inorganic and organic compounds [115]. The application of composite materials, which incorporate both organic and inorganic components, has recently attracted more attention. Combining the materials results in several beneficial properties that the separate materials could not produce. For instance, the elasticity and easy processing of polymers and mechanical properties of inorganic constituents are integrated [116]. 

Being an amino polysaccharide, chitosan contains several OH and NH_2_ groups that might serve as coordination and reaction sites. The fact that chitosan is made from chitin, the second-most abundant natural polymer after cellulose, makes it an extremely abundant and inexpensive material. Chitosan’s use as a material in real world applications is, nevertheless, constrained by its weak mechanical characteristics, which can be overcome via crosslinking with high mechanical strength materials, such as zeolites [116]. Khanday WA et al. synthesized composite beads by cross-linking chitosan and zeolite derived from oil palm ash and labeled it as Z-AC/C [117]. The activated oil palm ash was first hydrothermally treated before being beaded with chitosan. The effect of weight percentage of chitosan and Z-AC on the adsorption of MB (methylene blue) and AB-29 (acid blue-29) dyes was studied. Increase in the adsorption of AB29 and decrease in MB adsorption was observed by increasing the percentage of chitosan and vice versa. The Z-AC/C composite with 50:50 weight ratios of chitosan and zeolite well-adsorbed both AB-29 and MB dyes and was used for further studies. For the Z-AC/C composite, the adsorption capacities at 30 °C, 40 °C, and 50 °C were 212.76 mg/g, 238.09 mg/g, and 270.27 mg/g for AB29, 151.51 mg/g, 169.49 mg/g, and 199.20 mg/g for methylene blue, and 212.76 mg/g, 238.09 mg/g, and 270.27 mg/g for acid blue-19, respectively [117].

pH plays a crucial role in adsorption processes as the surface charge, speciation, and degree of ionization of adsorbate are influenced by the pH of the solution. pH influence on the adsorption of AB29 and MB on the Z-AC/C composite was studied in the pH range from 3–13 with 100 ppm initial dye concentration at 30 °C. It was observed that at a pH from 3–5, adsorption of AB29 was great and decreased linearly at pH 13. In the case of MB, the reverse phenomena were reported. At a pH from 3–5, adsorption of MB was less and linearly increased up to a certain pH and stayed constant afterward. At a low pH, electrostatic attractions between the adsorbent’s negatively charged surface and the positively charged H^+^ ions of AB29 dye are responsible for faster adsorption. As the pH of the solution increases, the hydrogen ions get diminished, lowering the attraction between the dye molecule and composite resulting in decreased adsorption of AB29. In case of MB, at a low pH, the adsorption is low due to the competition between MB and protons at binding sites. At a higher pH, the surface attains a negative charge, hence, the adsorption increases. Similarly, the initial dye concentration effect on the adsorption was also examined and it was observed that low concentrations reached equilibrium faster than high concentrations in the case of both dyes; the number of unoccupied active sites per dye molecule is low and the motion of dye molecules toward binding sites gets hindered [117].

Pizarro et al. prepared a composite using natural zeolite (NZ) and commercial cationic polymer, Polyammonium cation (SC—581), and labeled it as MZ (Modified zeolite) [118]. The material was investigated for adsorption of sulphate, a pollutant originating from processes of sulphate mining. Modification of zeolite with cationic polymer induces a positive charge on the adsorbent surface, which helps to bind sulphate ions. Also, the positive charge of MZ remains intact above pH 4, while the NZ holds the negative charge. The adsorption capacities of NZ were almost doubled with polymer impregnation. The effect of ion strength was also investigated, indicating that the adsorbent functions well below a KCl concentration of 0.050 mol/liter [118].

**Figure 17:**
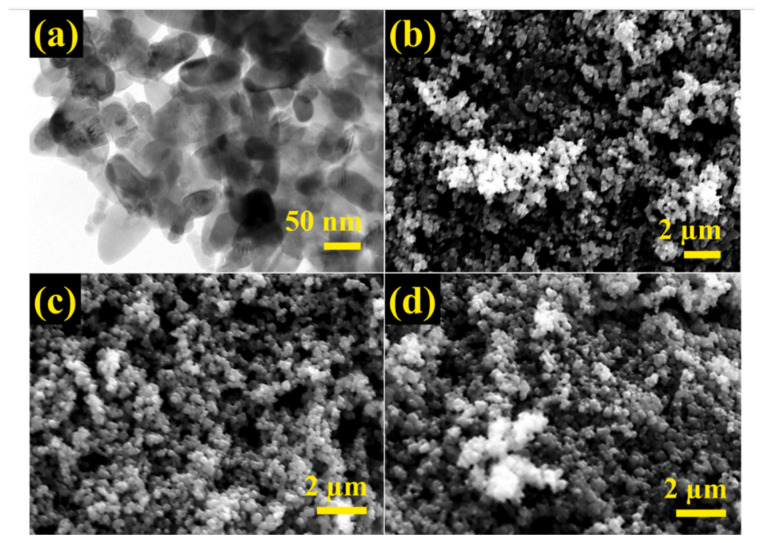
(**a**) TEM images of PPy/Ze NCs; SEM images of (**b**) PPy/Ze NCs, (**c**) PPy/Ze NCs after RB adsorption, (**d**) PPy/Ze NCs after RR adsorption. Reprinted with permission from Ref. [25]. Copyright 2022 Elsevier.

Senguttuvan et al. synthesized the zeolite/polypyrrole composite for the removal of reactive blue and reactive red dyes [25]. By performing oxidative polymerization of pyrrole in the presence of zeolite, the composite nano-adsorbent was fabricated. The FE-SEM and TEM images of PPy/Ze composites are shown in Figure 17. The particles are mostly agglomerated with a spherical shape and an average particle size between 40 and 80 nm. Even after adsorption of RB and RR, the PPy/Ze nanocomposites’ morphology did not change, which indicates the uniform dye adsorption on the surface of PPy/Ze nanocomposites.

With a 1.8 mg/mL catalyst dosage, and 75 ppm initial dye concentration, the nanocomposite adsorbed 88.3% of RR and 86.2% of RB within 75 min. Interaction between the dyes and PPy/Ze NCs was mostly mediated by aromatic, hydroxyl, and amide functional groups. The adsorption mechanism involved hydrogen bonding and electrostatic interactions. Senguttuvan et al. also reported the same PPy/Ze NCs for 83.5% removal of Cr(VI) in 50 min [119].

Yigit et al. fabricated beads of natural composite material using clinoptilolite, a natural zeolite, and alginate, a naturally occurring polymer and labeled it A-C (alginate-clinoptilolite) [120]. These A-C beads were employed in the mixture of heavy metals carrying copper (Cu^+2^), lead (Pb^+2^), and cadmium (Cd^+2^) ions. For 10 ppm of initial metal concentration, a constant operation with 2 mL/min flow rate revealed 98% of lead uptake. The repeatability test revealed that the efficiency of the adsorbent was up to the mark until 3 cycles with regeneration via HNO_3_ washing [120].

Conducting polymers has attracted a lot of interest because of their appealing features, including electrical conductivity and optical qualities. Due to its fascinating qualities, such as excellent chemical stability, cost-effectiveness, and facile synthesis, Polyaniline (PANI) is a unique and amazing polymer of the conducting polymer family. Additionally, upon visible light excitation, PANI can donate the electrons and act as a good hole transporter [121,122]. Hence, in addition to being adsorbent, PANI can also act as a photocatalyst and degrade organic pollutants.

To make zeolite/conducting polymer-based (nano-)composites, four alternative routes can be used [123]. (i) The organic solvent is enclosed in the zeolite cavities first; subsequently, oxidative polymerization is performed to produce polymeric chains in the zeolitic cavities [124]. (ii) Zeolites containing oxidant ions, such as Fe(III) and Cu(II) are reacted with monomer and acid vapors [125]. (iii) In the presence of zeolite, by performing in situ polymerization of the monomer, polymers can be developed inside or outside the channels of zeolite [126,127]. (iv) Powdered zeolite and conducting polymer are mixed mechanically [128]. Methods (ii) and (ii) of the previously outlined techniques hold the most interest since they produce nanoscale polymeric chains that are embedded in zeolite cavities. Therefore, as the polymeric chains are arranged at the nanoscale, the mechanical, electrical, chemical, and optical characteristics may be enhanced [123].

Abukhadra et al. synthesized the heulandite/polyaniline composite for the efficient removal of light green SF (LGSF), methylene blue (MB), and Congo red dyes from water [129]. Abukhadra et al. prepared the heulandite/polyaniline composite by the mechanical mixing of heulandite, natural zeolite, and synthesized conducting polymer, polyaniline. According to Tauc plot calculations, the optical band gaps of HU/PANI and PANI were 1.69 eV and 2.98, respectively. To examine the photocatalytic activities, studies were carried out both in dark conditions and with artificial visible light. The pseudo-second order and the Elovich model both provided good fits for the kinetic results. The Langmuir isotherm model provided a good description of the adsorption process in the dark, and the estimated q_max_ was 44.6 mg/g. The experimental data were well-fitted to the Freundlich and Temkin models under visible light illumination compared to the Langmuir model. This shows the function of photodegradation by the HU/PANI composite in improving the multilayer adsorption. The quadratic programming projected that the ideal conditions for the highest elimination percentage in the dark, i.e., 70.9%, 5.5 ppm dye concentration, pH 3, 24 mg dosage of Hu/PANI, and contact duration of 430 min. Whereas, in the presence of visible light, at 15 mg catalyst dosage, 15 ppm dye concentration, contact time of 589 min, pH 3, and 97% dye removal is possible [129].

**Figure 18:**
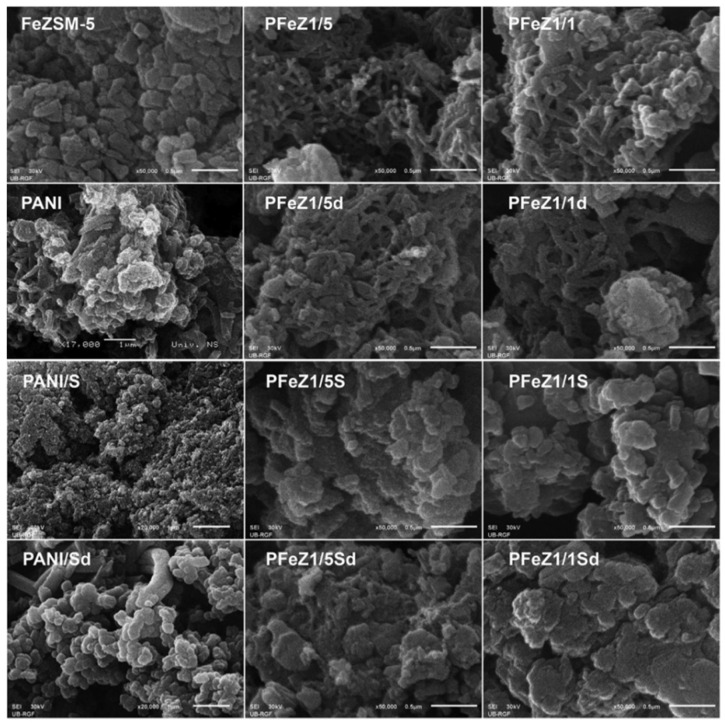
SEM images of PANI, FeZSM-5, and their composites. Reprinted with permission from Ref. [33]. Copyright 2018 Elsevier.

Milojević-Rakić investigated the polyaniline/FeZSM-5 composite for the removal of glyphosate, a herbicide via oxidative degradation [33]. Different weight ratios of aniline/FeZSM-5 was used. The method employed for synthesis was the oxidation polymerization of FeZSM-5 with ammonium peroxy disulphate as an oxidant, with and without using acid (H_2_SO_4_). 1/1 and 1/5 weight ratios of aniline/FeZSM-5 were used to make the composites. The composite with the 1/1 and 1/5 ratios of aniline/FeZSM-5 and synthesized without using acid was labeled as PFeZ1/1 and PFeZ1/5, respectively. Similarly, the composite with the 1/5 ratio of aniline/FeZSM-5 and synthesized using acid was labeled as PFeZ1/1S and PFeZ1/5S, respectively. The NH_4_OH treated (deprotonated) forms of PFeZ1/1, PFeZ1/5, PFeZ1/1S, and PFeZ1/5S were labeled as PFeZ1/1d, PFeZ1/5d, PFeZ1/1Sd, and PFeZ1/5Sd. Polyaniline was also synthesized and treated similarly and labeled as PANI, PANI/S, PANId, and PANIS/d, the same way as composites. The morphological analysis can be seen in Figure 18.

By performing the conductivity analysis, it was observed that the composites synthesized with the use of acid have higher conductivity in comparison to composites synthesized without acid. Additionally, the decrease in conductivity was observed by increasing the loading of zeolite; this elucidates the lower conducting nature of zeolites. Moreover, with the help of thermogravimetric analysis, the weight content of polyaniline and FeZSM-5 in the composites were evaluated. Table 7 gives the weight content of PANI and FeZSM-5 as well as the percentage degradation and removal of glyphosate from the solution [33].

The composites prepared without added acids showed better removal and degradation than those synthesized via adding acids. The deprotonation significantly reduced the degradation and removal efficiencies of all catalysts. From the data given in the above Table 7, it can be concluded that composite PFeZ1/5 showed the higher removal efficiency among all other catalysts. The PFeZ1/5 has an increased number of iron sites; hence, an increased amount of degradation is observed [33].

Milojevic’-Rakic et al. also prepared the ZSM-5/polyaniline composite by glyphosate adsorption [26]. The ZSM-5/polyaniline composite was prepared by performing the oxidative polymerization of aniline with and without added acid. Similar to the above-reported studies, the deprotonation of samples was also conducted. Among all the adsorbents, the deprotonated form of PANI prepared via acid synthesized polyaniline showed the maximum adsorption of glyphosate, i.e., (98.5 mg/g). The decrease in adsorption capacities was observed by increasing the zeolite loadings. The poor adsorption properties of ZSM-5 and composites containing the high weight ratios of ZSM-5 were most likely caused by its high microporosity, which is unfavorable for adsorption of comparatively huge glyphosate molecules. The linear, regular, and defect-free emeraldine base structure of PANI/Sd chains results in effective interaction with the glyphosate molecules. This was attributed to superior glyphosate adsorption characteristics of PANI/ Sd [26].

## 3. Comparison of Removal Efficiencies

The brief literature survey on zeolite and its composites for wastewater treatment is compiled in Table 8.

## 4. Conclusions, Challenges, and Future Perspectives

Zeolites are the materials widely used as adsorbents and ion-exchangers for the remediation of pollutants. This study describes the synthesis, removal process, mechanism, and application of zeolite-based materials in wastewater treatment, which gives first-hand information for researchers who want to explore zeolite-based materials. 

Researchers have doped various materials, such as metal oxides, polymers, and carbon-based materials, with zeolites to make zeolite-based composites. In most cases, these composites exhibit higher removal efficiency than bare zeolites. Additionally, the composites also enable zeolites to function as an effective photocatalyst for the total breakdown of contaminants, which is noticeably less apparent in bare zeolites. Composites of zeolite with semi-conducting materials, such as semiconducting metal oxides, graphene, CNTs, and conducting polymers, are of great interest because of the photodegradation of the contaminants.

The low efficiency, lack of homogeneous properties, lack of accessibility, and high levels of impurities in natural zeolite led to the synthetic preparation of zeolites. Major challenges are faced in the synthesis of zeolites, such as high-cost, the tedious and time-consuming synthesis and filtration process, generation of alkaline wastewater, etc. Research needs to be conducted for the synthesis of zeolites with homogenous properties, cost minimization, and easy processibility in order to establish the material for its easy application on the industrial level. 

The raw natural zeolites, i.e., pristine zeolites without any modifications, have a negative charge across its framework and are only capable of attracting cationic contaminants. Some modifications expand its application for the elimination of anionic pollutants as well. Zeolites can also be modified through doping with foreign materials.

This review motivates researchers to further investigate the photocatalytic behavior of zeolites and zeolite-based materials, since very few studies have been undertaken for the estimation of degradation pathways, contribution of ROS species (scavenger studies), and calculation of band edge positions. In future research, these aspects should also be considered for the better understanding of photodegradation in zeolite-based materials. The photocatalytic devices of zeolite-based materials for wastewater treatment are still not available in the market. Future interest should be on the preparation of zeolite-based wastewater purifiers that work on both the concept of photocatalysis and adsorption, which are easy to synthesize and can be realized in a much cheaper way so they can be easily commercialized and become feasible on an industrial scale. 

## Figures and Tables

**Table 1:** Pollutants, their sources, adverse effects, and removal techniques.

Sr. No.	Type of Pollutant	Examples	Sources	Adverse Effects	Removal Techniques	Ref.
1	Suspended solids and sediments.	Sand particles, eroded minerals, etc.	Mining, coal washing, etc.	Reduced light penetration in water, temperature change, etc.	Gravity settling, centrifuge, etc.	[38,39]
2	Oxygen demanding wastes.	Dissolved organic matter.	Domestic wastes, pulp and paper mill, wastes from food processing plants, animal sewage, slaughter houses, agricultural runoffs, etc.	Reduce the oxygen levels in water.	Carbon oxidation process, co-aggulation, fluctuation.	[50]
3	Organic dyes.	Methylene blue, methyl orange, crystal violet, rhodamine B, etc.	Textile, pharmaceutical, food, laser printing industries.	Diarrhea, breathing difficulty, nausea, vomiting, gastrointestinal issues, carcinogenic and oncogenic effects.	Adsorption, membrane separation, advanced oxidation processes (AOPs), biological decolorization.	[2,51,52]
4	Pesticides.	Organochlorines such as DDT, chlordane, aldrin, organofluorines such as PFOA, PFOS.	Agricultural activities.	Respiratory and skin conditions, cancer, reproduction issues, endocrine disruption, etc.	Advanced oxidation processes (AOPs), activated sludge treatment, adsorption, membrane technologies, etc.	[53,54]
5	Heavy metals.	Zinc (Zn), mercury (Hg), lead (Pb), arsenic (As), iron (Fe), cadmium (Cd), etc.	Surface run-off, roadworks, metal-based industries, automobiles, etc.	Haze, corrosion, eutrophication, and can lead to acid rain, also inhibits the biodegradation of organochlorines.	Adsorption, ion exchange.	[55,56]
6	Radioactive pollutants.	Caesium, strontium, uranium etc.	Nuclear power plants	DNA damage, thyroid, lung and skin cancers, hair loss etc.	Precipitation, distillation, microbial remediations, etc.	[3]
7	Nutrients.	Nitrogen, phosphorus, etc.	Agricultural fertilizers, run-offs from storm water, household detergents, human wastes, etc.	Eutrophication, reduction in oxygen level of water, reduced sunlight penetration, affect the growth of plants, etc.	To remove phosphorus biological nutrient removal (BNR) and nitrification and denitrification to remove to remove nitrogen.	[57]
8	Human, animal, and plant pathogens (pathogen microorganisms).	Different types of bacteria, viruses, protozoa, and helminths.	Animal and human fecal wastes, household and laundry wastewater.	Waterborne diseases such as polio, hepatitis cholera, anemia, typhoid, gastroenteritis, etc.	Natural elimination by temperature or prolonged life or adsorption to particles and sedimentation disinfection by chlorination and UV radiation.	[58,59,60]

**Table 2:** Name and chemical formula of some natural zeolites. Reprinted with permission from Ref. [15]. Copyright 2010 Elsevier.

Name of Zeolite	Chemical Formula
Clinoptilolite	(Na_2_, K_2_ Ca)_3_Al_6_Si_30_O_72_·21H_2_O
Phillipsite	K_2_(Na_2_, Ca)_2_Al_8_Si_10_O_32_·12H_2_O
Mordenite	(Na_2_, Ca)_4_Al_8_Si_40_O_96_·28H_2_O
Stilbite	Na_2_Ca_4_Al_10_Si_26_O_72_·30H_2_O
Chabazite	(Na_2_, Ca, K_2_)_2_Al_4_Si_8_O_24_·12H_2_O
Analcime	Na_16_Al_16_Si_32_O_96_·16H_2_O
Ferrierite	(Na_2_, K_2_, Ca, Mg)_3_Al_6_Si_30_O_72_·20H_2_O
Laumontite	Ca_2_Al_8_S_16_O_48_·16H_2_O
Scolecite	Ca_4_Al_8_Si_12_O_40_·12H_2_O
Heulandite	Al_8_Si_28_Ca_4_O_68_·24H_2_O

**Table 3:** Classification of zeolites according to their pore size.

Class of Zeolite	Number of Rings	Free Pore Diameter (nm)
(a) Zeolites with small pores	8	0.3 to 0.45
(b) Zeolites with medium pores	10	0.45 to 0.6
(c) Zeolites with large pores	12	0.6 to 0.8
(d) Zeolites with extra-large pores	14	0.8 to1.0

**Table 4:** Classification of zeolites considering Si to Al ratio.

Class of Zeolite	Range of Si:Al Ratio
(e) Zeolites with small Si:Al ratio	1.0 to 1.5
(f) Zeolites with intermediate Si:Al ratio	2 to 5
(g) Zeolites with large Si:Al ratio	10 to several thousands

**Table 5:** Classification of zeolites as per their structure type [64].

Framework Type Code	Symmetry	Channel Dimensionality	Framework Density (Å^3^)	Total Volume (Å^3^)	Accessible Volume (%)	Order	Reference Material
ANA	Cubic	3D	19.2 T/1000	2497.2	0.00	Fully ordered	Analcime
BEA	Tetragonal	3D	15.3 T/1000	4178.4	20.52	Partially disordered	Beta polymorph A
CHA	Trigonal	3D	15.1 T/1000	4178.4	20.52	Fully ordered	Chabazite
DFT	Tetragonal	3D	17.7 T/1000	451.7	6.58	Fully ordered	DAF-2
ERI	Hexagonal	3D	16.1 T/1000	2239.5	15.10	Fully ordered	Erionite
FAU	Cubic	3D	13.3 T/1000	14,428.8	27.42	Fully ordered	Faujasite
FER	Orthorhombic	2D	17.6 T/1000	2051.3	10.01	Fully ordered	Ferrierite
HEU	Monoclinic	2D	17.5 T/1000	2054.8	9.42	Fully ordered	Heulandite
LAU	Monoclinic	1D	18.0 T/1000	1333.6	9.57	Fully ordered	Laumontite
MFI	Orthorhombic	3D	18.4 T/1000	5211.3	9.81	Fully ordered	ZSM-5
MOR	Orthorhombic	2D	17.0 T/1000	2827.3	12.27	Fully ordered	Mordenite
MRE	Orthorhombic	1D	19.7 T/1000	2442.5	6.55	Partially disordered	ZSM-48
NAT	Tetragonal	3D	16.2 T/1000	1231.5	9.06	Fully ordered	Natrolite
PHI	Orthorhombic	3D	16.4 T/1000	1953.7	9.89	Fully ordered	Phillipsite

**Table 6:** Modification of zeolites, their advantages and disadvantages. Reprinted from Ref. [91].

Modification Method	Process	Advantages	Disadvantages	Ref.
Acid/base treatment.	Simple ion exchange using dilute acid solution.	Pore volume and electrostatic surface area is increased.	Decrease in CEC due to dealumination, decrease in thermal stability.	[92]
Surfactant modification.	By introducing cationic organic surfactants such as tetramethylammonium, hexadecyl trimethyl ammonium (HDTMA), n-cetyl pyridinium (CPD), etc.	Increases the hydrophobicity of zeolite, making it appropriate for the adsorption of a wide range of organic pollutants.	Complicated functional groups are formed for cationic exchange sites due to formation of admicelle.	[93,94,95]
Ultrasonic modification.	By sonicating with a solvent with help of an ultrasonicator bath.	Impurities are removed from the channel and the surface area is increased.	Always used in combination with other methods, inefficient.	[96]
Thermal modification.	By heating in the oven or muffle furnace.	Evaporation of water, removal of contaminants from the channel, and expansion of the pore diameter.	Uneven heating.	[97]

**Table 7:** Weight contents, percentage removal, degradation, and adsorption in composites. Reprinted with permission from Ref. [33]. Copyright 2018 Elsevier.

Sample	Weight Content		FeZSM/PANI Weight Ratio	% Removal	% Degradation	% Adsorption
	FeZSM-5	PANI				
PANI	-	-	-	12.4	11.6	0.8
ZSM-5	-	-	-	12.8	7.1	5.7
PFeZ1/1	43.1	52.2	0.83	40.8	31.9	8.9
PFeZ1/5	77.9	18.1	4.30	80.4	66.5	13.9
PANI/S	-	-	-	10.2	9.7	0.5
PFeZ1/1S	42.2	51.6	0.82	26.8	22.6	4.2
PFeZ1/5S	76.9	16.8	4.58	18.1	13.6	4.5
PANId	-	-	-	56.6	54.4	2.2
PFeZ1/1d	47.4	48.4	0.98	13.6	8.4	5.2
PFeZ1/5d	78.9	15.0	5.26	20.4	14.6	5.8
PANI/Sd	-	-	-	6.6	2.8	3.8
PFeZ1/1Sd	47.2	48.4	0.98	7.4	1.1	6.3
PFeZ1/5Sd	79.4	15.5	5.12	3.8	0	3.8

**Table 8:** Comparison of effluent removal efficiencies of zeolites and zeolite-based composites.

Material	Source /Synthesis Approach	Band Gap (eV)	Contaminant	Mechanism	Dosage (mg/mL)	Concentration (ppm)	Contact Time	Removal	Light Source	Published Year	Ref
Zeolite	Indonesia commercial		NH_4_^+^	Adsorption, ion exchange	0.00152	12.9	134.89 min	98%		2020	[76]
Natural zeolite	Chinese commercial		NH_4_^+^	Adsorption	0.048	80	180 min	96%		2010	[11]
Natural zeolite	Australia commercial		Methylene blue	Adsorption	0.25	3.55	200 h	6.8 × 10^−5^ mol/g		2005	[75]
Natural zeolite	Australia commercial		Rhodamine B	Adsorption	0.25	3.55	50 h	2.1 × 10^−5^ mol/g		2005	[75]
Hydrogenated form of natural zeolite	Carranco Blanco	2.63	Caffeine	Photocatalysis	10	50	4 h	99%	UV	2020	[13]
Synthetic zeolite	Hydrothermal treatment using aluminum iso propoxide	3.29	Methylene blue	Photocatalysis	2	10	180 min	85%	UV	2020	[14]
Titania- Supported zeolite	In situ using TiCl_4_ impregnation	3.31	Methylene blue	Photocatalysis	0.33	30	60 min	40%	UV	2010	[130]
Titania- supported zeolite	In situ using TiCl_4_ impregnation	3.31	Direct blue 71	Photocatalysis	0.33	30	60 min	55%	UV	2010	[130]
Titania- supported zeolite	In situ using TiCl_4_ impregnation	3.31	Direct yellow 8	Photocatalysis	0.33	30	60 min	62.5%	UV	2010	[130]
Heulandite/ Polyaniline/nickel oxide	In situ polymerization followed by Ni_2_O_3_ impregnation	1.42	Safranin T	Photocatalysis	0.35	5	1 min	100%	Solar irradiation	2018	[30]
bentonite/ PANI/Ni_2_O_3_	In situ polymerization followed by Ni_2_O_3_ impregnation	1.61	Safranin-O	Photocatalysis	0.5	5	90 min	100%	Sunlight	2018	[131]
Heulandite/ polyaniline	Mechanical mixing	1.69	LGSF	Photocatalysis	0.3	15	589 min	97%	VIS	2018	[129]
Heulandite/ polyaniline	Mechanical mixing	1.69	MB	Photocatalysis	0.2	20	589 min	68.77%	VIS	2018	[129]
Fe-Al bimetallic oxide loaded zeolite	Co-precipitation		Cr(IV)	Adsorption	40	20	300 min	84.9%		2020	[106]
Zeolite/ZnO	Wet impregnation method		Caffeine	Adsorption/photocatalysis	500	25	120 min	100%	UV	2018	[24]
Zeolite/ZnO	Co-precipitation		Pb(II)	Adsorption	3	100	30 min	92%		2017	[101]
Zeolite/ZnO	Co-precipitation		As(V)	Adsorption	3	10	30 min	85.7%		2017	[101]
Zeolite/TiO_2_	Sol-gel		Sulfadiazine	Photocatalysis	1	1	120 min	93.31%	UV	2018	[23]
Zeolite/Activated carbon	Hydrothermal		Cu^+2^	Adsorption	2	240	60 min	92.8%		2020	[19]
Zeolite/Activated carbon	Hydrothermal		Rhodamine B	Adsorption	2	240	60 min	94.2%		2020	[19]
Zeolite/Activated carbon	Fusion/Hydrothermal		Methylene blue	Adsorption	1	100	30 h	83%		2016	[114]
Zeolite/Poly pyrrole	In situ polymerization		Reactive Red	Adsorption	1.8	75	75 min	88.3%		2022	[25]
Zeolite/Poly pyrrole	In situ polymerization		Reactive Blue	Adsorption	1.8	75	75 min	86.2%		2022	[25]

## Data Availability

Not applicable.
